# Morphology of Inner Retina in Rhesus Monkeys of Various Ages: A Comparative Study

**DOI:** 10.1155/2019/7089342

**Published:** 2019-03-03

**Authors:** Jingfei Chen, Qihui Luo, Chao Huang, Wen Zeng, Ping Chen, Qi Gao, Bing Chen, Wentao Liu, Lingzhen Pan, Zhengli Chen

**Affiliations:** ^1^Laboratory of Animal Disease Model, College of Veterinary Medicine, Sichuan Agricultural University, Chengdu, Sichuan, China; ^2^Key Laboratory of Animal Disease and Human Health of Sichuan Province, College of Veterinary Medicine, Sichuan Agricultural University, Chengdu, Sichuan, China; ^3^Agriculture Service Center of Baisha, Jiangjin, Chongqing, China; ^4^National Experimental Macaque Reproduce Laboratory, Ya'an, Sichuan, China

## Abstract

**Purpose:**

To investigate the changes of thickness in each layer, the morphology and density of inner neurons in rhesus monkeys' retina at various growth stages, thus contribute useful data for further biological studies.

**Methods:**

The thickness of nerve fiber layer (NFL), the whole retina, inner plexiform layer (IPL), and outer plexiform layer (OPL) of rhesus monkeys at different ages were observed with hematoxylin and eosin (H&E) staining. The morphology and the density of inner neurons of rhesus monkey retina were detected by immunofluorescence.

**Results:**

The retina showed the well-known ten layers, the thickness of each retinal layer in rhesus monkeys at various ages increased rapidly after infant, and the retina was the thickest in adulthood, but the retinal thickness stop growing in senescent. Quantitative analysis showed that the maximum density of inner neurons was reached in adolescent, and then, the density of inner neurons decreased in adults and senescent retinas. And some changes in the morphology of rod bipolar cells have occurred in senescent.

**Conclusions:**

The structure of retina in rhesus monkeys is relatively immature at infant, and the inner retina of rhesus monkeys is mature in adolescent, while the thickness of each retinal layer was the most developed in the adult group. There was no significant change in senescence for the thickness of each retinal layer, but the number of the neurons in our study has a decreasing trend and the morphological structure has changed.

## 1. Introduction

The structural and functional complexity of retina makes it vulnerable to alterations throughout the animal life. The morphology and function of retina may change along with the age. Optical coherence tomography (OCT) has indicated that nerve fibers, ganglion cells, and the inner plexiform layers are thinner in diabetic retinopathy (DR) patients [1–3]. Retinal thinning, ganglion cell layer (GCL) loss, and changes in specific layers are also described in other neurological disorders, including multiple sclerosis, schizophrenia, and Parkinson's disease (PD) [4–7]. Retinal degenerative diseases in humans are incurable. Thus, to search for effective treatments, a number of animal models have been developed in order to mimic different human retinal diseases.

The retina of rodent models has provided an invaluable tool to study the morphology and function for more than 30 years. Among these animal models, they are mainly focused upon secondary changes affecting inner retinal cells at various times [8] or morphology of cells in the ganglion cell layer [9]. In primate models, the emergence and differentiation of the IPL were analyzed in the embryonic rhesus monkey [10, 11]. Dacey has studied the morphology of ganglion cells in the rhesus monkey [12], and quantitative survey of bipolar and amacrine cell types in the inner nuclear layer in marmoset retina was reported [13]. Rhesus monkeys, closest to human in the genetic background, have been used for developmental, physiological, and anatomical behavioral analysis of vision. However, the morphology of the inner retina at various stages in primates during the postnatal period was not well defined.

In this study, rhesus monkeys were selected as objects to investigate the structure of rhesus monkey retinas of various ages and provide a detail analysis of the changes occurring during development. This study aims at (1) investigating the changes of each layer in the retina of rhesus monkeys at various ages and (2) observing the morphology and survival of inner retinal cells at various ages, e.g., RGCs and second-order neurons in the retina of rhesus monkeys.

## 2. Materials and Methods

### 2.1. Subjects

In this study, 20 healthy rhesus monkeys used for this study were maintained by the National NHPs Research Center of PriMed Shines Biotech Co., which is located in Ya'an, Sichuan, Southwest China. These monkeys were divided into four groups (*n* = 5 monkeys per group) according to age [14], including infant group, adolescent group, adult group, and senescent group. The selections of sex were random in our study, and food and water were provided *ad libitum*. All subjects underwent standard ophthalmologic examinations including refraction, orthoptic examination, IOP measurement, and slit-lamp examination. Participants had no known neurological disease, diabetes, or active cardiovascular disease. Animal welfare was conducted under the regulation of the National Institutes of Health Guide for the Care and Use of Laboratory Animals, issued by the Ministry of Science and Technology of China [15]. All efforts were made to minimize suffering and the number of animals used in the study. Furthermore, these animals did not receive medical or immunomodulatory treatments prior to the experiment (Table 1).

### 2.2. Histological Slide Preparation

All animals were deeply anesthetized with 15 mg/kg of ketamine and sacrificed, the eyes were quickly enucleated and dissected, and the eye cups were placed in formaldehyde-acetic acid-alcohol (FAA) for 24 hours. The right eyes for H&E and the left eyes for immunofluorescence were paraffin embedded, and serial sections (5 *μ*m) through the optic nerve head were processed using a microtome (2035 Biocut Leica).

### 2.3. Immunofluorescence

The samples are preincubated in 10% normal goat serum in PBS containing 1% bovine serum albumin (BSA) and 0.3% Triton X-100 for one hour at room temperature to block nonspecific binding activity. Then, the samples were incubated overnight (4°C) with anti-NeuN antibody (Abcom Plc, Cambridge, UK), anti-pkc-*α* antibody (Abcom Plc, Cambridge, UK), or anti-parvalbumin antibody (Bioss, Beijing) to immunolabel RGCs, rod bipolar, or amacrine, respectively. Then, the sections were incubated with secondary Alexa Fluor 488 for 1.5 hours at room temperature, and DAPI was used for nuclei staining. Sections were washed, mounted, and imaged by fluorescence microscopy (Olympus BX43, Japan). PBS was treated as negative control.

### 2.4. Retinal Morphometry Observation and Image Analysis

**Figure 1:**
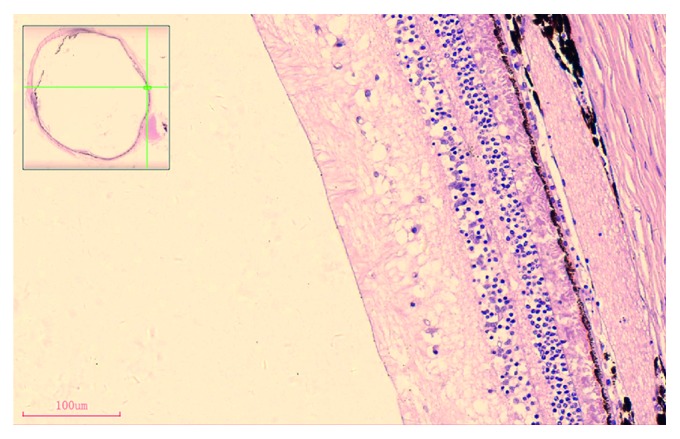
Images of a local section of an adult rhesus monkey retina stained with H&E by brightfield microscopy showing the predefined region for further morphometric analysis, and the region was defined as 1/3 of the total linear length of the superior nasal side of the retina from the optic nerve head; bar = 100 *μ*m.

Sections of the retina tissue stained by H&E and immunofluorescence were analyzed, and the images were captured by brightfield microscopy (Olympus BX51, Japan) to observe retinal structures. The sections were analyzed at the superior nasal side of the central retina defined as the region one-third along the length of the retina starting from the optic nerve outwards to the periphery for further morphometric analysis (Figure 1). Morphometric analysis was utilized to quantify the changes, including the thickness of the retinal nerve fiber layer (NFL), the whole retina that defined as the total thickness of pigment epithelium and retinal sensory layer, and the density and size of RGCs. Briefly, thickness was measured using the Jiangsu Jetta 801 morphological image analysis system (Jieda, Nanjing, China), and the density of RGCs was counted from the sections. For each animal, cells were counted within the upper central regions from three sections.

### 2.5. Statistical Analysis

Statistical analysis was presented as mean ± standard deviation (SD) using IBM SPSS Statistics (version 20.0; IBM Crop.; Armonk, New York, NY). Statistical analysis was performed using one-way ANOVA. *P* value ≤ 0.05 was considered statistically significant (^*∗*^*P* < 0.05, ^*∗∗*^*P* < 0.01).

## 3. Results

**Figure 2:**
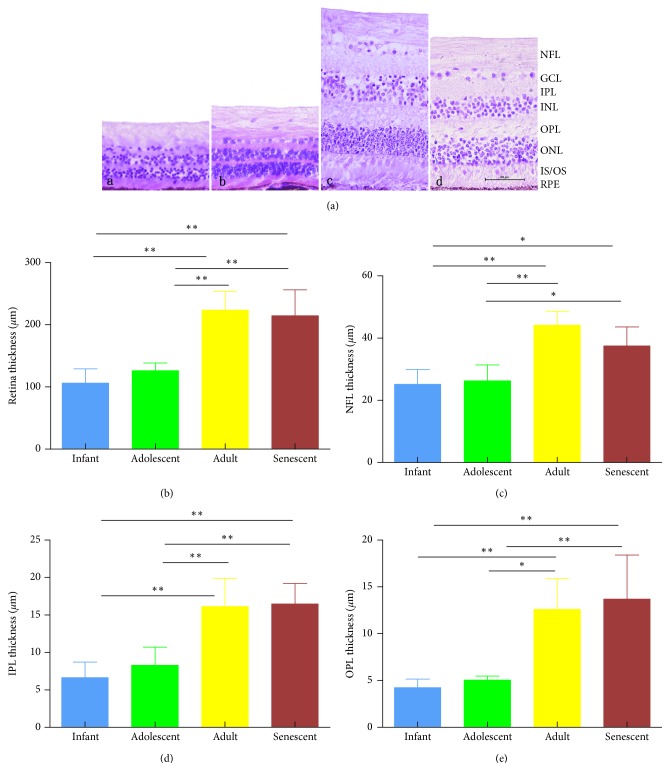
(a) Photograph of rhesus monkey retina stained with H&E at the infant (a), adolescent (b), adult (c), and senescent (d) stage, respectively; bar = 50 *μ*m. (b) Graph comparing the mean thickness (mean ± SDs) of the whole retina in each group. (c) Average NFL thickness of rhesus monkeys at different ages. (d) The thickness of IPL in rhesus monkeys at different ages. (e) Statistical analysis of NFL thickness in rhesus monkeys of various ages. NFL, nerve fiber layer; GCL, ganglion cell layer; IPL, inner plexiform layer; INL, inner nuclear layer; OPL, outer plexiform layer; ONL, outer nuclear layer; IS, inner segment; OS, outer segment; RPE, retinal pigment epithelium. Results were presented as mean ± SDs (*n* = 5). ^∗^Significant difference between groups, *P* ≤ 0.05.

The effects of age on retinal morphology were examined by H&E staining in rhesus monkeys (Figure 2(a)). The retina showed the well-known ten layers, as described earlier in human, and the retina of the rhesus monkey forms three distinct cell body layers, separated by two plexiform layers [16]. The outer plexiform layer (OPL) between inner nuclear layer (INL) and outer nuclear layer (ONL) was gradually broadened. The IPL and OPL showed obvious reticular structure, and the nuclei in the INL and ONL become larger and denser at the adolescent stage, as compared with that in the infant group.  Table 2 shows the thickness of each retinal layer in rhesus monkeys at various ages. The thickness of NFL (Figure 2(c)) in the central retinal region revealed that there were no differences between infant and adolescent subjects. However, the thickness of NFL in adults was significantly (*P* < 0.01) thicker than that in the adolescent group. No statistical difference in the thickness of NFL was found between adults and the senescent. The above results suggested that the thickness of NFL changed obviously from adolescent to adulthood and showed that it was thickest in the adult group, but we did not find a significant change of the thickness of NFL in the senescent compared with adults. The total retinal thickness (Figure 2(b)) revealed that there were no differences between infant group and adolescent group. However, there was a significant (*P* < 0.01) increase in the adult group, compared with the adolescent group. The mean thickness of the whole retina in the adult group had no significant difference compared with aged rhesus monkeys. The thickness of IPL (Figure 2(d)) and OPL (Figure 2(e)) in each group showed almost the same trend as the thickness of NFL and the whole retina. The results suggested that the thickness of each retinal layer in rhesus monkeys at various ages increased rapidly after adolescent and approached the thickest in adulthood, but stopped growing after adults.

**Figure 3:**
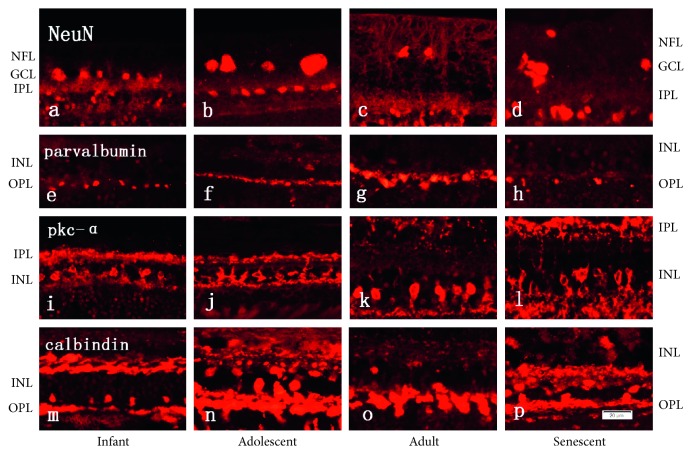
Sections of eyes from rhesus monkey retinas at the infant, adolescent, adult, and senescent, respectively. a–d: cells were stained with NeuN antibody to immunolabel RGCs; e–h: the morphology of amacrine cells was revealed by parvalbumin; i–l: anti-pkc-*α* antibody was used to immunolabel rod bipolar cells in the INL; m–p: sections were stained with calbindin antibody to sign horizontal cells. NFL, retinal nerve layer; GCL, ganglion cell layer; IPL, inner plexiform layer; INL, inner nuclear layer; OPL, outer plexiform layer; *n* *=* 5 for each group; bar = 20 *μ*m.

**Figure 4:**
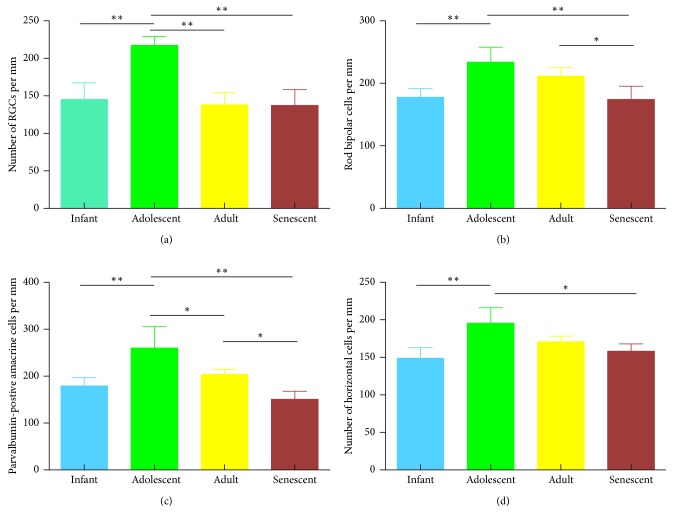
(a) A graphical summary of the density of RGCs cell in retinas from rhesus monkeys at infant, adolescent, adult, and senescent. (b) Histograms illustrating the density of rod bipolar cells at different stages. (c) A graph comparing the density of parvalbumin-positive amacrine cells at each stage. (d) A graph comparing the density of horizontal cells stained by calbindin in retinas from rhesus monkeys of various ages (*n* = 5). Results were presented as mean ± SDs. ^*∗*^Significant difference between groups, *P* <0.05..

The RGCs were labeling with NeuN antibody (Figure 3), and the morphology of RGCs in the rhesus monkey was dominated by midget and parasol cells. The results suggested that the mean density of RGCs tended to decrease (*P* < 0.01) from infant to adolescent (Figure 4(a)). Similarly, the mean density of RGCs in the retina of adult rhesus monkeys is significantly (*P* < 0.01) decreased when compared with the adolescent group. In addition, it is also found that the density of RGCs in the retina of senescent rhesus monkeys is significantly (*P* < 0.01) lower when compared with adolescent rhesus monkeys. However, no significance was found between adult and senescent rhesus monkeys.

The morphological structure of rod bipolar cells [14], amacrine cells, and horizontal cells was displayed by immunofluorescence staining with anti-PKC-*α* antibody, anti-parvalbumin antibody, and anti-calbindin antibody, respectively, in the INL [17] (Figure 3). The three proteins expressed predominately in the INL and normally distributed over the retinal surface with a typical center-to-periphery gradient. Rod bipolar cells in the infant, adolescent, and adult groups were arranged neatly and exhibited the well-known morphology with elliptical cell bodies, bushy and a long axon and chandelier-like dendritic arborization as described before [8]. However, in the senescent group, neurite regeneration sprouted from the top of rod bipolar cell indicated that the function of the second-order rod bipolar cells transfer signal was affected. The density of amacrine in the adolescent group was the largest compared with other groups and formed a clear horizontal line. The dendrites of horizontal cells developed better in the adolescent group compared with other groups. Quantitative analysis showed that the maximum density of parvalbumin-positive amacrine cells was reached significantly (*P* < 0.01) at adolescent in the INL; then, the density of all amacrine cells decreased significantly in adult and senescent retains (Figure 4(d)). Findings were the same in the rod bipolar cells stained by PKC-*α* (Figure 4(c)), and the difference between adolescent and adult groups was not significant. Finally, the density of horizontal cells (Figure 4(d)) in adolescent rhesus monkeys stained with anticalbindin is significantly (*P* < 0.05) larger when compared with the infant and senescent groups.

## 4. Discussion

The present study documented morphological changes in the retina at various ages through the whole life span in rhesus monkeys. Clinically, OCT is used for the quantitative detection of retinal thickness, which is widely used due to its noninvasive and contactless imaging method [18]. In the current OCT technology, it is not possible to reliably differentiate the boundary between GCL and IPL [19]. However, the advantage of our study is that the structure of each layer of the retina can be clearly identified by H&E staining. Previous data have suggested that the central inner retina around the area of high acuity (AHA) changed triphasically which thickened in the embryonic stage, thinned transiently after birth, and then resumed thickening [20]. Statistical findings of retinal thickness in the present study show that developmental changes in the thickness of the retinal layer are not showing a linear relationship with age. In our study, no obvious change in the thickness of the retina was found between the infant and the adolescent. The thickness increased rapidly after youth and was the thickest in adulthood, but we did not find any decline in senescent. With age, alterations of the thickness of IPL, OPL, and retinal thickness in each group were consistent with NFL. NFL, IPL, and OPL are important positions of the synapses and axons of inner neurons and play an important role in forming the network system, so synapses and axons of inner neurons could be well developed in adulthood. Rhesus monkeys have about 1–1.5 million RGCs. [21] Over 17 types distinguished, the midget, parasol, and small bistratified cell populations form the large majority of RGCs [22, 23]. The density of RGCs in our study revealed a statistically significant increase with the development of the retina from the infant to the adolescent and a reduction at adult. However, whether the change in the density of RGCs is due to the apoptosis or retinal expansion for local densities is unknown. Amacrine cells, bipolar cells, and horizontal cells are the second-order neurons in the retinal circuitry that play a crucial role in visual function. All amacrine cells labeled with parvalbumin are postsynaptic to rod bipolar cells and are important neurons that drive rod information to the cone bipolar pathway by means of conventional chemical synapses [17, 24]. In our study, the dendrites of horizontal cells are basically the same as those of bipolar cells. With age, horizontal cells which normally make triad synaptic connections with photoreceptors and bipolar cells may have dendrites that extended into the ONL in aged retinas, and these were spatially juxtaposed with the elongated dendrites of bipolar cells, which are previous data [25]. Some studies suggested that inner retinal damage may be more susceptible to hyperglycemia than in outer retinal neurons, which is characterized by the substantial reduction in amacrine cells and RGCs density [26, 27]. In our research, cell density among groups showed obviously different, same as discussed in previous studies about capuchin monkey [28]. The morphology and quantity of second-order neurons varied with age, the dendrites were well developed, and the density of the cells got to the highest at the adolescent stage.

In conclusion, certain morphological changes represented in this article have a clear physiological correlate. With age, the eye went through certain changes, including the thickness of each layer, the density, and the morphology of RGCs and second-order neurons. The maximum density of second-order neurons was reached at adolescent in the INL, while synapses and axons of inner neurons were well developed in adulthood for each layer and got to the thickest in adulthood. The above results indicate that the structure of the retina in rhesus monkeys is relatively immature at infant, and the inner retina of rhesus monkeys is mature in adolescent, while the thickness of each retinal layer was the most developed in the adult group. There was no significant change in senescent for the thickness of each retinal layer, but the number of the neurons in our study has a trend of decreasing and the morphological structure has changed. These changes may lead to more visual clarity and more complete development from infant to adolescent, but vision acuity may decline gradually from adult to senescent.

## Figures and Tables

**Table 1:** The detailed information of experimental animals (*n* = 5).

Group	Ages (years)	Weight (kg)
Infant	1.26 ± 0.11	1.19 ± 0.15
Adolescent	3.82 ± 0.42	3.40 ± 0.31
Adult	14.16 ± 0.96	8.11 ± 1.03
Senescent	20.6 ± 0.69	9.70 ± 1.63

**Table 2:** The thickness of each retinal layer in rhesus monkeys at various ages (*n* = 5).

Group	Retina	NFL	IPL	OPL
Infant	106.02 ± 23.05	25.16 ± 4.75	6.64 ± 2.09	4.24 ± 0.92
Adolescent	125.84 ± 12.53	26.26 ± 5.13	7.97 ± 2.85	5.03 ± 0.42
Adult	223.35 ± 30.19	44.15 ± 4.46	22.79 ± 9.5	21.25 ± 11.74
Senescent	214.57 ± 41.64	37.47 ± 6.14	43.14 ± 7.55	31.08 ± 12.29

## Data Availability

The data used to support the findings of this study are available from the corresponding author upon request.
